# Cryptic lncRNA-encoded ORFs: A hidden source of regulatory proteins

**DOI:** 10.1172/JCI167271

**Published:** 2023-03-01

**Authors:** Anindya Dutta, Hui Li, Roger Abounader

**Affiliations:** 1 Department of Genetics, The University of Alabama at Birmingham, Birmingham, Alabama, USA.; 2 Department of Pathology, University of Virginia, Charlottesville, Virginia, USA.; 3 University of Virginia NCI-Designated Comprehensive Cancer Center, Charlottesville, Virginia, USA.; 4 Department of Microbiology, Immunology and Cancer Biology and; 5 Department of Neurology, University of Virginia, Charlottesville, Virginia, USA.

## Abstract

A majority of the human genome is transcribed into noncoding RNAs, of which long noncoding RNAs (lncRNAs) form a large and heterogeneous fraction. While lncRNAs are mostly noncoding, recent evidence suggests that cryptic translation within some lncRNAs may produce proteins with important regulatory functions. In this issue of the *JCI*, Zheng, Wei, and colleagues used an integrative functional genomic strategy to systematically identify cryptic lncRNA-encoded ORFs that play a role in estrogen receptor–positive (ER^+^) breast cancer (BC). They identified 758 cryptic lncRNA-encoded ORFs undergoing active translation, of which 28 had potential functional and clinical relevance in ER^+^ BC. The LINC00992-encoded polypeptide GT3-INCP was upregulated in ER^+^ BC and drove tumor growth. GT3-INCP was regulated by estrogen and the ER and acted via the transcription factor GATA3 to regulate BC susceptibility and risk genes. These findings discern a largely unexplored class of molecules and have implications for many pathologies, including cancer.

## Functionally and clinically relevant cryptic lncRNA-encoded proteins

While most of the genome is transcribed into RNA, only about 2% of these RNAs are translated into functional proteins and peptides. The remaining, roughly 98%, is made up of several classes of noncoding RNA molecules (1). Among these classes, long noncoding RNAs (lncRNAs) are a large and heterogeneous group of noncoding RNAs longer than 200 nucleotides. Individual lncRNAs were discovered in the early 1990s, but their identification as a new class of noncoding regulatory molecules occurred much later with the characterization of the transcriptional landscape of the mammalian genome (2). Over 50,000 human lncRNAs have been identified (3). lncRNAs regulate gene expression at transcriptional, posttranscriptional, and epigenetic levels (4). While lncRNAs are mostly noncoding, recent evidence suggests that cryptic translation within some lncRNAs may produce proteins with important regulatory functions. However, a better and more comprehensive knowledge of the functions and mechanisms of action of cryptic lncRNA-encoded proteins is still lacking.

A recently published study in the *JCI* by Zheng, Wei, et al. (5) partly fills the above-mentioned knowledge gap by using an integrative functional genomic strategy to systematically identify cryptic lncRNA-encoded ORFs that play a role in estrogen receptor–positive (ER^+^) breast cancer (BC). They also investigated in further depth the function and mechanism of action of one of them. The authors first identified 758 cryptic lncRNA-encoded ORFs undergoing active translation in ER^+^ BC using ribosome profiling (ribo-seq). They then used a CRISPR/Cas9-based knockout screen of the encoded proteins/polypeptides to assess the effects of encoded proteins and polypeptides on cell fitness. By integrating the screen data with TCGA RNA-seq data of deregulated cryptic lncRNA-encoded proteins in luminal BC, they identified 28 cryptic ORFs with potential functional and clinical relevance in ER^+^ BC. Of these, they chose to focus on the LINC00992-encoded cryptic ORF because the lncRNA expression associates with poor prognosis in luminal BC.

## Cryptic LINC00992-encoded GT3-INCP in BC

LINC00992 is an intergenic lncRNA that is best known for roles unrelated to the encoded protein. Zheng, Wei, et al. first determined its full transcript and identified a 5′ extension that corresponded to an active translation site of a polypeptide, GATA3-interacting cryptic protein (GT3-INCP), based on ribo-seq data and subsequent antibody-based detection. They showed that GT3-INCP localized primarily to the nucleus and that it was upregulated in ER^+^ BC cell lines and tumors. Using loss-of-function and gain-of-function approaches, they demonstrated that GT3-INCP had tumor-promoting functions in vitro and in vivo. To uncover the molecular mechanism underlying GT3-INCP’s oncogenic effects, they used affinity purification followed by mass spectrometry to identify proteins that interacted with GT3-INCP. Among the interacting proteins that displayed changes in expression between luminal tumor and normal tissue was GATA3, a transcription factor that is essential to the establishment and maintenance of luminal epithelial cell identity during mammary gland development and that is frequently mutated in BC. GATA3 is also a marker of ER^+^ primary luminal BC tumors. Using rescue experiments, the authors showed that the interaction between GT3-INCP and GATA3 was important for mediating the tumor-promoting function of GT3-INCP. RNA-seq and gene set enrichment analyses revealed that GT3-INCP and GATA3 coregulated a common expression program impacting the genes associated with estrogen response and cell proliferation. They then used ChIP-seq to identify the direct targets of GT3-INCP and found that half of the targets overlapped with GATA3 targets on the chromatin. Using an integrated analysis of RNA-seq and ChIP-seq data in ER^+^ BC cells, together with TCGA data, the authors identified the common direct targets of GT3-INCP/GATA3 that are important for mediating their tumor-promoting function. Among these targets were two BC susceptibility and risk genes, *MYB* and *PDZK1*. Notably, GT3-INCP was upregulated by estrogen and ER and was important for estrogen-dependent cell growth and estrogen-regulated gene expression.

## Conclusions

Zheng, Wei, et al. (5) demonstrated that lncRNAs are a source of mostly unknown and hidden proteins and polypeptides that can play important regulatory roles in cancer. The authors convincingly showed that one of these proteins, GT3-INCP, was deregulated and acted as an important mediator of malignancy in ER^+^ BC. Uncovering these cryptic lncRNA-encoded proteins is important for a better understanding of physiological and pathological processes, including cancer, as well as for the identification of therapeutic targets.

The study was well conducted. The approach for the screening of cryptic lncRNA-encoded proteins provides a template for other investigators to use when searching for such proteins in other contexts. The identification of GT3-INCP using an antibody to complement the ribo-seq finding confers validity to the ribo-seq finding. Of note is that one definition of a lncRNA is that it should not encode a peptide beginning with an initiator methionine (M) of more than 50 amino acids. Adhering to this definition, if the full 5′ extension of LINC00992 had been identified at the time of annotation, the 120 amino acid long ORF starting with an initiator ATG would not have been annotated as a lncRNA. Indeed, a 131 amino acid or a 120 amino acid peptide, derived from the same locus, is annotated in databases as a potential peptide: XP_047273942.1 (https://www.ncbi.nlm.nih.gov/protein/XP_047273942.1?report=GenPept) or EAW48932.1 (https://www.ncbi.nlm.nih.gov/protein/EAW48932.1). The dissection of the function and mechanism of action of GT3-INCP was very well performed and the data are convincing. While Zheng, Wei, et al. (5) convincingly show a role for GT3-INCP in ER^+^ BC, the broader impact of cryptic lncRNA-encoded proteins remains unclear. There are up to 50,000 unique lncRNAs, but only 758 cryptic lncRNA-encoded ORFs with an ATG start codon were identified by Ribo-TISH. This finding suggests that only a small fraction of lncRNAs encodes proteins. In addition, another screen could be added to determine what fraction of the total pool of lncRNAs is associated with ribosomes, because that will establish whether a candidate lncRNA is efficiently translated into a protein (like conventional mRNAs), or whether it occasionally and accidentally is translated into a micropeptide. Because lncRNA expression can be tissue and pathological condition specific, it is possible that additional lncRNAs that encode proteins are expressed in other tissues or other cancers. In addition, the efficiency with which a lncRNA is translated into a peptide may vary by tissue or pathology, so such studies should extend to different lineages and different cancers. The tissue and pathological specificity of cryptic lncRNA-encoded proteins could make them exquisite targets for therapies by avoiding the potential side effects associated with broad expression patterns that many proteins possess. Interestingly, while GT3-INCP was primarily found in the nucleus, it was also partially detected in the cytoplasm. Many lncRNAs are found in both subcellular compartments where they exert different functions (6, 7). The authors unraveled the nuclear function and mechanism of action of GT3-INCP but did not investigate its cytoplasmic role.

This work provides an insight into a largely unexplored class of molecules, with implications for the understanding and therapy of many pathologies, including cancer.

## Version 1 03/01/2023

Electronic publication
